# Reflections on RCOT campaign ‘complexity of occupation’: The dimensions of occupation and the dimensions of practice

**DOI:** 10.1177/03080226251369709

**Published:** 2025-08-27

**Authors:** Daniel Cezar da Cruz

**Affiliations:** Department of Rehabilitation and Health Professions, School of Health, Leeds Beckett University, Leeds, UK

The Royal College of Occupational Therapists (RCOT) campaign on the ‘Complexity of Occupation’, part of its Workforce Strategy, promotes occupation-centred practice as the profession’s distinct contribution to health and social care. Research shows this approach strengthens professional identity, enhances health outcomes and clarifies roles (Phillips et al., 2023). Fisher and Marterella (2019) describe occupation-centred practice as a profession-specific way of thinking that keeps occupation at the heart of reasoning. Translating theory into action requires therapists to apply occupational knowledge meaningfully (Hitch and Pepin, 2020). This editorial reflects on these ideas and explores practical ways to embed the dimensions of doing, being, belonging and becoming into occupational therapy.

In the RCOT lecture ‘Doing, being, belonging and becoming applied to occupational therapy practice’, I presented how Wilcock and Hocking (2015) clearly described these dimensions and their implications for health. In this editorial, key reflections from the RCOT lecture will be discussed, providing the occupational therapy workforce with considerations for transforming occupation-centred practices. Next, I will present how the dimensions of occupation relate to another dimension: the dimensions of practice, with practical applications.

The Oxford University Press (2011) defines a dimension as ‘an aspect or feature’ (p. 402) of a situation. In this context, we are discussing the extent or depth to which the dimensions of doing, being, belonging and becoming can be applied in occupational therapy practice. The dimensions of practice are therefore based on (1) reflection, (2) formulating questions and (3) taking action at various levels of practice, as inspired by the dialogue proposed by Freire (1970). The concept of the dimensions of practice is needed because it is recognised that occupational therapists face challenges in their daily practice, particularly when balancing managerial roles, generic tasks or working in settings that may not prioritise occupation-centred approaches. However, understanding the dimensions of practice provides hope and reassurance to start making changes in some dimensions. Occupational therapists need not feel fearful or guilty for not always being fully occupation-centred. Instead, by starting with some of these dimensions, they can initiate a process of change and gradually move towards more occupation-centred practice.

Alternatively, the concept of dimensions of practice can be applied from different perspectives. Table 1 presents a structure designed to guide and encourage individual reflections and apply the dimensions of occupation:

**Table 1.** Practising the dimensions of practice using the framework of doing, being, belonging and becoming.

Dimensions of occupation	Dimensions of Practice		
	Reflection	Questions	Actions
Doing			
Being			
Belonging			
Becoming			

It’s built on three pillars: reflection, questioning and action. It acknowledges that we may not always be fully occupation-centred, but it offers a pathway to move closer to that ideal. To make these dimensions realistic, it is essential to navigate the complexities of real-world occupational therapy. The evidence has shown that occupation-centred practice is guided by theory and philosophy of occupation, language and documentation, understanding and incorporating the person’s occupation and context into practice – including assessment, goal setting and intervention (Ford et al., 2021). Applying this evidence into practice, if taking into account the occupational therapy process, and the occupational therapist competencies (Bossers et al., 2008), in multidisciplinary teams (MDT) interactions, occupational therapists can seek for opportunities to discuss the concepts of doing, being, becoming and belonging using examples from their caseloads. When building collaborative relationships with service users, these dimensions should create space for dialogue and reflection, fostering deeper engagement and understanding of occupational beings.

In addition, occupational therapists can reflect, question and act by locating what dimensions of practice are possible in their settings. This can be achieved by including questions about *doing, being, belonging* and *becoming* in their non-standardised assessments or identifying what standardised assessments or outcome measures can inform people of *doing, being, belonging* and *becoming*. In the intervention, they can reflect on how the things they do are focused or not on the person’s *doing, being, belonging* and *becoming* and make changes to be truly occupation-centred. For example, they can question at which level others are included to support the person in participating and engaging in occupations: family members, relatives, neighbourhood, community and other local resources (belonging). This translation cannot occur without professional reasoning. Within the Occupational Pan Paradigm (Hitch and Pepin, 2020), occupational therapists can apply various Conceptual Models of Practice – such as the Model of Human Occupation (MOHO), the Canadian Model of Occupational Performance and Engagement (CMOP-E), the Person-Environment-Occupation-Performance, the Kawa (River) and many others – to creatively articulate the dimensions of *doing, being, belonging* and *becoming*.

Regarding language, occupational therapists can reflect on how effectively they adapt their communication to service users, families, carers, the multidisciplinary team (MDT) and other stakeholders to enable them to understand the concepts of *doing, being, becoming* and *belonging*. They can ask how these terminologies might be tailored to describe an individual’s circumstances in documentation or records. Therapists may also consider whether the MDT can comprehend these nuanced concepts. However, if we, as occupational therapists, are expected to understand the medical and technical terminology used by other health and social care professions, should there not also be reciprocity, respect and recognition for the language that defines our professional identity?

Continuing professional development is essential in a dynamic profession. Occupational therapists can embrace change, reflect critically and grow through communities of practice. Activities such as supervision, education and peer engagement build professional identity and connection (French and Clarke, 2024; Phillips et al., 2023). These actions align with RCOT’s four pillars: Professional Practice, Facilitation of Learning, Leadership and Evidence, Research and Development (Royal College of Occupational Therapists, 2022); therefore, the dimensions of occupation and the dimensions of practice are not restricted to clinical practice. I conclude this article with a reflective question to initiate changes: to what extent does your occupational therapy practice process reflect an occupation-centred approach and what steps can you take to improve it? The answer now is in your reflections, questions and actions.
